# The Complex Work of Proteases and Secretases in Wallerian Degeneration: Beyond Neuregulin-1

**DOI:** 10.3389/fncel.2019.00093

**Published:** 2019-03-20

**Authors:** Marta Pellegatta, Carla Taveggia

**Affiliations:** Division of Neuroscience and INSPE at IRCCS San Raffaele Scientific Institute, Milan, Italy

**Keywords:** wallerian degeneration, remyelination, secretases, matrix metalloproteinases, fibrinolysis

## Abstract

After damage, axons in the peripheral nervous system (PNS) regenerate and regrow following a process termed Wallerian degeneration, but the regenerative process is often incomplete and usually the system does not reach full recovery. Key steps to the creation of a permissive environment for axonal regrowth are the trans-differentiation of Schwann cells and the remodeling of the extracellular matrix (ECM). In this review article, we will discuss how proteases and secretases promote effective regeneration and remyelination. We will detail how they control neuregulin-1 (NRG-1) activity at the post-translational level, as the concerted action of alpha, beta and gamma secretases cooperates to balance activating and inhibitory signals necessary for physiological myelination and remyelination. In addition, we will discuss the role of other proteases in nerve repair, among which A Disintegrin And Metalloproteinases (ADAMs) and gamma-secretases substrates. Moreover, we will present how matrix metalloproteinases (MMPs) and proteases of the blood coagulation cascade participate in forming newly synthetized myelin and in regulating axonal regeneration. Overall, we will highlight how a deeper comprehension of secretases and proteases mechanism of action in Wallerian degeneration might be useful to develop new therapies with the potential of readily and efficiently improve the regenerative process.

## Peripheral Nerve Regeneration

In the peripheral nervous system (PNS), nerves can be damaged following various events such as traumatic injuries, metabolic alterations and genetic peripheral neuropathies. All these events significantly impact the quality of life of both patients and caregivers. Regardless of the nature of the triggering cause, after injury both Schwann cells and neurons are compromised as their reciprocal interdependence determines that damaging one cell type will eventually influence the other. Unlike the central nervous system, the PNS possesses the intrinsic capability to regenerate, as axons can regrow over long distances to reach their final target and Schwann cells are able to remyelinate them. Nonetheless, the regenerative process often remains incomplete, due to the inability of the repair machinery to maintain an extended growth promoting response (Gaudet et al., 2011).

After traumatic injury, PNS axons can regrow and remyelinate following a process termed Wallerian degeneration. The cascade of events leading to the axon regrow and the re-establishment of a functional myelinated axo-glia unit has been originally described by August Waller as the result of nerve axotomy (Waller and Owen, 1850), and might partially differ from those observed after crush injury (Stoll et al., 2002; Gaudet et al., 2011; Rotshenker, 2011). In this review article, we will report the role of molecules and modifiers of the extracellular matrix (ECM) in the process of regeneration and remyelination. Since these molecules have been investigated in both models of crush injury and axotomy we will generally refer to their role in peripheral nerve injury.

During early Wallerian degeneration, axons degenerate and Schwann cells adopt a specific response to injury: the repair program (Gerdts et al., 2016). Specifically, denervated Schwann cells lose their myelin sheath and downregulate the expression of myelin-associated genes, among which Egr2/Krox20, MPZ, MBP, MAG and periaxin. In addition, they start to proliferate and upregulate molecules characteristic of their immature stage such as L1, NCAM, p75NTR and GFAP (Jessen and Mirsky, 2008). However, after injury, Schwann cells do not simply revert their phenotype to an earlier developmental stage, but undergo a proper “trans-differentiation” phase in which they acquire unique characteristics, becoming true “repair cells” (Arthur-Farraj et al., 2012; Jessen et al., 2015). In particular, they upregulate neurotrophic factors supporting axon survival and elongation, a necessary step towards regeneration (Fontana et al., 2012; Brushart et al., 2013). In addition, they specifically activate a massive myelin-breakdown and play an early pivotal role in removing myelin debris, which represents a barrier to the regrowth of axons distal to the injury site (Brown et al., 1994; Perry et al., 1995; Hirata and Kawabuchi, 2002; Gomez-Sanchez et al., 2015; Jang et al., 2016). During the first week after injury, repair Schwann cells also produce cytokines that induce macrophages’ activation and recruitment from the bloodstream (Bolin et al., 1995; Kurek et al., 1996; Shamash et al., 2002; Tofaris et al., 2002; Roberts et al., 2017). In the following stages, indeed, macrophages assume a primary role in debris removal and growth factors production, supporting axons regeneration and Schwann cells migration through the distal nerve stump (Gaudet et al., 2011; Gerdts et al., 2016). Within this complex pro-regenerative environment, repair Schwann cells adopt an elongated bipolar morphology and align to form regenerative tracks from the injury site to the nerve target, called “Bungner bands” (Gomez-Sanchez et al., 2017). These structures generate along the basal lamina that previously surrounded the Schwann cell-axon unit in intact nerves and support injured axons regrowth through the distal stump to reach their final target.

Axo-glial communication is essential for nerve repair: not only Schwann cells create a permissive environment for axons regrowth, but also axon-derived signals promote differentiation of repair cells (Fawcett and Keynes, 1990; Stassart et al., 2018). The re-establishment of the axo-glial unit during later phases of the regenerative process allows Schwann cells to re-differentiate and remyelinate the newly generated axon and to re-establish rapid saltatory conduction of the nerve impulse. Nonetheless, remyelinated axons have shorter internodes and thinner myelin sheaths as compared to uninjured nerves (Fancy et al., 2011; Stassart et al., 2018).

Given the inefficiency in the regenerative process, there is an evident need to envisage more effective therapeutic strategies allowing complete recovery. Recent advances in biomaterial and biomedical engineering have provided new strategies to improve regenerative capabilities in injured nerves with synthetic conduits (Tajdaran et al., 2018). These scaffolds support the reconstruction of long nerve gaps, as they allow the application of exogenous agents with neurotrophic properties. Although not pertinent to this review, nerve-scaffolding techniques represent a strategic line of research in nerve regeneration.

Finally, axonal loss and repeated attempts of nerve regeneration are key features also of several peripheral neuropathies, like Charcot Marie Tooth hereditary neuropathies (Stassart et al., 2018). Thus, the development of more adequate treatments to promote nerve repair could similarly benefit these pathologies for which there is no effective therapy.

## Neuregulin-1

Neuregulins are transmembrane proteins belonging to the family of epidermal growth factor (EGF)-like growth factors (Falls, 2003). They signal by binding to the tyrosine kinase receptors of the ErbB family expressed on Schwann cells (Yarden and Sliwkowski, 2001), thereby activating different intracellular signal transduction pathways in glial cells (Yang et al., 2004; Gu et al., 2005). Neuregulin-1 (NRG-1) is the most important gene among the members of this family and it is also the best characterized. The *Nrg1* gene encodes for several isoforms, all containing an EGF-like domain, which is required to activate the ErbB receptors. All NRG1 isoforms differ mainly for the composition of their N-terminal end, on the base of which they have been classified into six different groups (Garratt et al., 2000; Buonanno and Fischbach, 2001; Mei and Nave, 2014). Distinct NRG1 isoforms use specific promoters to drive their expression in a tight spatio-temporal regulated pattern. Indeed, they are implicated in different cellular function, like migration, proliferation, morphogenesis and control of cell size (Buonanno and Fischbach, 2001; Esper et al., 2006; Britsch, 2007; Mei and Nave, 2014).

Among the various types of Neuregulins, we will focus on NRG1 type I and type III, which are the main isoforms thus far investigated in PNS development and regeneration. NRG1 type I is a single-pass membrane protein and presents an Immunoglobulin-like domain at the N-terminus, while NRG1 type III, in addition to the transmembrane domain, possesses a cysteine-rich domain in the N-terminal portion that further anchors the protein to the plasma membrane (Ho et al., 1995).

**Figure 1:**
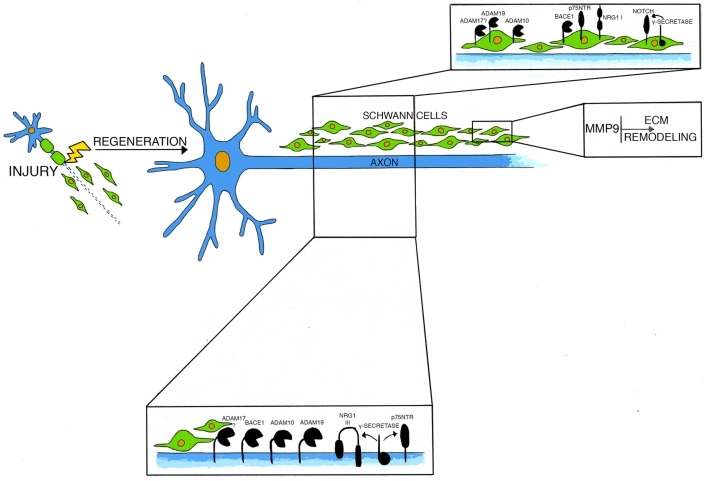
This model depicts molecules and secretases mainly involved in axonal regeneration after injury and their cellular localization.

In the PNS, NRG1 type III is essentially expressed on axonal membranes (Figure 1). During nerve formation, NRG1 type III is required for Schwann cells proliferation and differentiation (Nave and Salzer, 2006). Indeed, in mutant mice lacking NRG1 type III expression, Schwann cells are absent, and this causes a severe cell death of dorsal root ganglia (DRG) and motor neurons (Wolpowitz et al., 2000). During development, NRG1 type III forces Schwann cells to choose between myelination and non-myelination (Taveggia et al., 2005). Of note, axonal levels of NRG1 type III also determine the thickness of the myelin sheath: mice overexpressing axonal NRG1 type III are hypermyelinated, whereas mice haploinsufficient for NRG1 type III are hypomyelinated (Michailov et al., 2004; Taveggia et al., 2005). Binding of NRG1 to their cognate ErbB2/ErbB3 receptors, activates multiple signal transduction pathways in Schwann cells, including PI3K-AKT1, calcineurin and MAPK signaling pathways (Taveggia et al., 2005, 2010; Kao et al., 2009; Pereira et al., 2012). Of note, NRG1 type III activity is regulated by the extracellular cleavage of secretases (Willem, 2016). While the beta secretase β-site amyloid precursor protein-cleaving enzyme 1 (BACE1) activates NRG1 type III, enhancing myelination (Hu et al., 2006; Willem et al., 2006), cleavage of NRG1 type III by the α-secretase ADAM17 inhibits myelination (La Marca et al., 2011; Bolino et al., 2016). Accordingly, transgenic mice lacking BACE1 are hypomyelinated (Hu et al., 2006; Willem et al., 2006) and mice depleted of neuronal ADAM17 are hypermyelinated, with a phenotype that remarkably resembles NRG1 type III overexpressing mice (Michailov et al., 2004;La Marca et al., 2011).

Following traumatic nerve injury, the expression of several components of the NRG1 signaling machinery is dys-regulated (Birchmeier and Bennett, 2016; Morano et al., 2018). For example, ErbB2/ErbB3 receptors expression is increased after damage and their activation status is enhanced (Cohen et al., 1992; Carroll et al., 1997; Kwon et al., 1997; Guertin et al., 2005), although the effective role of ErbB receptors in peripheral nerve injury is partly controversial (Atanasoski et al., 2006). Moreover, neuronal NRG1 type III expression decreases as soon as the Schwann cells-axon unit is destroyed but it is subsequently re-expressed during late phases of nerve regeneration, when axons re-innervate their target (Bermingham-Mcdonogh et al., 1997; Stassart et al., 2013).

Axonal NRG1 plays an important role in peripheral nerve repair, which, under many aspects, is different from that accomplished during nerve development (Birchmeier and Bennett, 2016). After an injury insult, axonal NRG1 is a driving force for regeneration and it controls the expression of genes involved in peripheral nerve injury (El Soury et al., 2018). Its juxtacrine interactions with Schwann cells, in fact, prompt both remyelination and normal re-innervation of neuromuscular junctions (Fricker et al., 2011). Accordingly, remyelination in mice lacking NRG1 expression in a subset of motor and sensory neurons is severely impaired (Fricker et al., 2011). Unexpectedly, these defects are transient, and axons in mutant nerves are efficiently remyelinated during late nerve regeneration (Fricker et al., 2013). NRG1 therefore is an important factor in regulating nerve repair following injury but, unlike development, it does not control the myelinating fate of axons.

While the above-mentioned studies suggest that axonal NRG1 is dispensable for long term remyelination, it has also been reported that neuronal overexpression of NRG1 type I and type III isoforms facilitates nerve repair and improves remyelination after injury (Stassart et al., 2013). Although these findings seem contradictory, it is possible that gain and loss of function approaches are targeting different mechanisms. Lack of NRG1 after injury might in fact be compensated with time by other molecules, whereas its overexpression could promote Schwann cells re-differentiation and favor nerve regeneration through a distinct process.

Unlike NRG1 type III, NRG1 type I expression is potently induced in Schwann cells early after nerve damage, and although dispensable for developmental myelination, it is critical for remyelination (Figure 1; Loeb et al., 1999; Falls, 2003; Stassart et al., 2013). Indeed, mutant mice specifically lacking NRG1 type I expression in Schwann cells poorly remyelinate after injury, suggesting that Schwann cells require autocrine NRG1 type I signaling to support re-differentiation and repair (Stassart et al., 2013).

Interestingly, administration of exogenous NRG1 type III and NRG1 type I promotes neurite outgrowth and nerve regeneration (Mahanthappa et al., 1996; Cai et al., 2004). Moreover, modulation of NRG1 activity in animal models of CMT1A (Fledrich et al., 2014), CMT1B (Scapin et al., 2018) and Congenital Hypomyelinating Neuropathy (Belin et al., 2018), can ameliorate the peripheral hypomyelination observed in these diseases, prevent axonal loss and promote nerve functional recovery, the latter all features observed also after nerve injury. Nonetheless, it has also been recently reported that NRG1 is already highly expressed in nerves of CMT1A pre-clinical models, thus indicating that delivery of exogenous NRG1 should be carefully evaluated (Fornasari et al., 2018).

Collectively these studies suggest that after damage, Schwann cells might have developed new strategies to accomplish many of their cellular functions. Thus, a better understanding of the molecular mechanism underlying their extraordinary plasticity remains crucial towards the development of powerful therapies leading to complete regeneration.

## BACE1

The beta secretase BACE1 is an aspartyl protease mostly involved in processing type I transmembrane proteins, which results in the generation of a cleaved, membrane-bound portion of the target molecule and in the release of a soluble fragment. BACE1 has been well characterized in the pathogenesis of Alzheimer’s disease, since it is one of the main enzymes responsible for the processing of the amyloid precursor protein and for the generation of amyloid beta plaques, a pathological hallmark of the disease (Sinha and Lieberburg, 1999; Vassar et al., 1999; Yan et al., 1999; Cai et al., 2001; Luo et al., 2001; Roberds et al., 2001).

Besides the amyloid precursor protein, BACE1 cleaves a variety of substrates (Hemming et al., 2009). Among them, in the nervous system, there are voltage-gated sodium channels (Wong et al., 2005; Kim et al., 2007, 2011; Gersbacher et al., 2010), the potassium channel proteins KCNE1 and KCNE2 (Hitt et al., 2012; Sachse et al., 2013), the adhesion molecules L1 and close homolog of L1 (CHL1; Hitt et al., 2012; Kuhn et al., 2012; Zhou et al., 2012), contactin-2 (Kuhn et al., 2012) and Seizure-related gene 6 protein (Sez 6; Kuhn et al., 2012). Processing of these substrates by BACE1 perturbs several brain functions, as these proteins are involved in different tasks. Indeed, BACE1 is crucial to generate axonal connections (Hitt et al., 2012), regulate axonal guidance (Kuhn et al., 2012; Zhou et al., 2012) and modulate neurite outgrowth and neural connectivity (Kuhn et al., 2012). Surprisingly, despite being implicated in processing many substrates, constitutive ablation of BACE1 in mice results in viable and fertile animals, with only moderate behavioral abnormalities (Cai et al., 2001; Luo et al., 2001; Roberds et al., 2001; Laird et al., 2005; Savonenko et al., 2008).

In the PNS, BACE1 is mainly expressed in motor neurons and in DRG sensory neurons (Willem et al., 2006) and its expression is highly similar to that of NRG1 type III. Nonetheless, BACE1 is present at very low levels also in Schwann cells in normal nerves and at higher levels in damaged nerves (Hu et al., 2015; Figure 1). Constitutive deletion of BACE1 results in severe myelination defects during development (Hu et al., 2006; Willem et al., 2006; Velanac et al., 2012). Interestingly, NRG1 cleavage is impaired in BACE1 null mice (Hu et al., 2006; Willem et al., 2006), and the hypomyelinating phenotype resembles that observed in NRG1 type III haploinsufficient mice (Michailov et al., 2004; Taveggia et al., 2005). Accordingly, in peripheral nerves, BACE1 directly cleaves axonal NRG1 type III, generating a membrane-tethered portion of the molecule that exposes the EGF-like domain allowing the activation of the ErbB2/ErbB3 receptors complex on Schwann cells, thereby promoting myelination (Hu et al., 2008; Velanac et al., 2012).

BACE1 is also implicated in peripheral nerve injury; specifically, in the absence of BACE1 remyelination is impaired. Accordingly, injured nerves of BACE1 null mice have thinner myelin sheath and increased number of un-myelinated axons (Hu et al., 2008). These defects have been ascribed to aberrant processing of axonal NRG1 type III, which impairs PI3K/AKT1 signaling pathway activity (Hu et al., 2008). More recently, also the contribution of glial BACE1, i.e., expressed in Schwann cells, has been investigated in nerve remyelination (Hu et al., 2015). Nerve graft experiments suggest that both axonal and Schwann cell-derived BACE1 activity are decisive for remyelination (Hu et al., 2015). Interestingly, since both BACE1 and NRG1 type I are induced in Schwann cells after injury (Hu et al., 2008, 2015; Stassart et al., 2013), it is likely that glial BACE1 contributes to nerve repair by promoting NRG1 type I cleavage (Hu et al., 2015), though this has never been formally demonstrated.

Other studies reported an opposite role for BACE1 in PNS remyelination. By analyzing early regenerative events, Farah et al. (2011) observed that BACE1 knockout mice present enhanced myelin and axonal debris clearance, an increased number of regenerated fibers and precocious re-innervation of neuromuscular junctions. These phenomena are likely due to neuronal BACE1, as overexpression of BACE1 in neurons determines a marked decrease in peripheral axons regeneration capacity (Tallon et al., 2017). These data suggest that neuronal BACE1 could act as a negative regulator of axonal regeneration in early events following injury, contradicting previous studies. To reconcile these conflicting results and to better clarify the role of BACE1 in nerve regeneration and remyelination, further studies in conditional knockout mice allowing ablation in defined temporal windows are necessary. Moreover, since BACE1 processes many substrates, which are expressed in different cell types and at specific stages of the repair process, the study of its cell autonomous role could help in defining the effector(s) through which BACE1 regulates specific stages of axonal regeneration and remyelination.

Since BACE1 has a prominent role in the generation of amyloid beta plaques during AD pathogenesis, a lot of effort has been devoted in developing small molecules that could inhibit the enzyme’s activity (Coimbra et al., 2018), however only one study has tested their efficacy in a model of nerve crush injury. A 7 days treatment with a BACE1 inhibitor starting immediately after damage increases regeneration and enhances myelin debris clearance (Farah et al., 2011), supporting the hypothesis that BACE1 inhibits early axonal regeneration (Farah et al., 2011; Tallon et al., 2017). Although the possibility of using BACE1 inhibitors to boost axonal regeneration is potentially promising, it is essential to better define their application, given the contradictory role of BACE1 after injury (Hu et al., 2008, 2015). Thus, further studies are required to exactly determine the efficacy of BACE1 inhibitor(s) in the resolution of PNS injury. Particularly relevant would be to define the exact time frame in which to administer these compounds to promote axonal growth without altering remyelination.

## A Disintegrin and Metalloproteinases

A Disintegrin And Metalloproteinases (ADAMs) are membrane-anchored metalloproteinases involved in the proteolytic processing of several transmembrane proteins and in the consequent regulation of the cleaved substrates’ activity (Schlöndorff and Blobel, 1999). ADAM proteases have both proteolytic and disintegrin characteristics (Schlöndorff and Blobel, 1999). They present a conserved structure composed by distinct domains (Schlöndorff and Blobel, 1999; Primakoff and Myles, 2000) and have been implicated in the release of several molecules among which EGF receptor ligands, tumor necrosis factor family members, neuregulins and other transmembrane proteins (Black and White, 1998; Blobel, 2000). ADAM proteins participate in many biological functions such as cell migration, cell membrane fusion and cytokines and growth factors shedding. Moreover, members of this family cooperate in muscle development, fertilization, neurogenesis, angiogenesis and cell fate determination (Schlöndorff and Blobel, 1999; Primakoff and Myles, 2000; Horiuchi et al., 2003; Seals and Courtneidge, 2003). Pathologies like inflammation-related disorders and cancer also implicate ADAM proteins (Seals and Courtneidge, 2003; Blobel, 2005).

It is thus not surprising that ADAM secretases have a role also in PNS myelination. In particular, the α-secretase ADAM17 possesses an intrinsic proteolytic activity, which inhibits NRG1 type III and the process of myelination during development. Thus, only a fine balance between activating and inhibitory signals coming from the activity of BACE1 and ADAM17 could physiologically regulate the correct formation of the myelin sheath (La Marca et al., 2011).

Not only ADAM17, but also ADAM10 and ADAM19, play important roles in PNS functioning (Figure 1). In particular, ADAM10 is highly expressed in Schwann-cells DRG neuronal co-cultures during myelination and its chemical inhibition impairs axonal outgrowth *in vitro* (Jangouk et al., 2009). Nonetheless, *in vivo* studies have shown that ADAM10 is dispensable for developmental myelination. In fact, both overexpression of a dominant negative form of the protein (Freese et al., 2009) as well as its conditional inactivation in a transgenic model (Meyer zu Horste et al., 2015) do not alter myelination. Interestingly, ADAM10 promotes axonal sprouting, as its deficiency in motor neurons impairs the extent of axons growth *in vitro* (Meyer zu Horste et al., 2015). Moreover, this secretase is critical for axonal outgrowth during peripheral nerve regeneration as deletion of ADAM10 in motor neurons reduces the axonal density of small caliber fibers after crush injury. Thus, ADAM10 seems mainly implicated in the specific control of post-traumatic outgrowth of axons (Meyer zu Horste et al., 2015).

ADAM19 is highly expressed in developing PNS, and its expression pattern resembles that of NRG1 (Kurisaki et al., 1998; Shirakabe et al., 2001); both proteins are co-expressed in DRG and in motor neurons (Marchionni et al., 1993; Shirakabe et al., 2001). Of note, ADAM19 can cleave membrane-anchored NRG1 proteins in cultured neurons (Kurisaki et al., 1998; Shirakabe et al., 2001; Wakatsuki et al., 2004). *In vivo* studies have shown that while ADAM19 is dispensable for PNS development, remyelination after nerve injury is impaired in its absence (Wakatsuki et al., 2009). Further, after injury, ADAM19 regulates the re-differentiation of Schwann cells from a pro-myelinating stage to a fully myelinating one by activating the AKT pathway (Wakatsuki et al., 2009).

Although ADAM10 and ADAM19 participate in the progression of nerve regeneration, nerve repair is eventually achieved in both mutants. It is thus possible that compensatory mechanisms intervene to favor remyelination, likely carried out by other secretases. Since ADAM17 is fundamental in nerve development and NRG1 processing, it would be important to assess the role of this secretase during nerve regeneration.

## Gamma Secretases

Gamma secretases (γ-secretases) are a multi-subunit protease complex responsible for the intramembrane cleavage of transmembrane proteins, mostly known for the generation of the β-amyloid peptide in Alzheimer’s disease (De Strooper et al., 1998; Wolfe et al., 1999). Beside the amyloid precursor protein, γ-secretases process several type I transmembrane proteins, among which the adhesion molecules N-cadherin and E-cadherin (Struhl and Adachi, 2000; Golde and Eckman, 2003), the neurotrophin receptor p75 (p75NTR; Jung et al., 2003), the β2 subunit of voltage-gated sodium channel (Kim et al., 2005), the protein receptor Notch (De Strooper et al., 1999) and Neuregulins (Dejaegere et al., 2008; Trimarco et al., 2014). Although no direct functional role of the γ-secretase complex has been described in peripheral nerve regeneration, some of its substrates are relevant for such process, in particular Notch and p75NTR.

Notch activity is regulated by a classical regulated intramembrane proteolysis (RIP) mechanism (Figure 1). After binding to its substrates, Notch is first cleaved in its extracellular domain (Pan and Rubin, 1997; Brou et al., 2000; Hartmann et al., 2002; van Tetering et al., 2009); this cleavage induces a subsequent process in the transmembrane domain, which is mediated by the γ-secretase complex (De Strooper et al., 1999; Struhl and Greenwald, 1999). Notch intracellular domain then translocates in the cell nucleus, where it regulates the transcription of genes important for cell development and differentiation (Kopan and Goate, 2000; Kopan and Ilagan, 2004; Louvi and Artavanis-Tsakonas, 2006). In peripheral nerves, Notch negatively regulates developmental myelination (Woodhoo et al., 2009). After injury, Notch is rapidly upregulated in peripheral nerves and, in a mouse model in which Notch is conditionally ablated in Schwann cells, the rate of Schwann cells dedifferentiation after axotomy is reduced (Woodhoo et al., 2009). This impairment relies on Notch intracellular domain, as a similar defect is found also in mice deficient in Schwann cells RBPJ (Recombination signal Binding Protein for Immunoglobulin Kappa J region), the key transcriptional mediator of canonical Notch intracellular signaling (Woodhoo et al., 2009).

p75NTR regulates a variety of cellular function in the nervous system, from programmed cell death, axonal growth and degeneration, to cell proliferation, myelination and synaptic plasticity (Kraemer et al., 2014; Meeker and Williams, 2015). This wide range of biological effects is due to the multiplicity of ligands and co-receptors which partner with p75NTR to regulate its signaling. In particular, p75NTR promotes cells survival through association with TRK receptors (Hempstead et al., 1991; Meeker and Williams, 2015; Pathak et al., 2018), inhibits axonal regeneration by interacting with Nogo receptor and Lingo (Wang et al., 2002; Wong et al., 2002; Mi et al., 2004) and induces apoptosis via high affinity binding with Sortilin (Friedman, 2000; Beattie et al., 2002; Nykjaer et al., 2004). Similarly to Notch, also p75NTR is regulated by RIP: upon ligand binding, the extracellular domain of the receptor is first cleaved by ADAM17 (Weskamp et al., 2004) and then processed by the γ-secretase complex, thereby releasing a small intracellular domain (Zampieri et al., 2005). p75NTR is highly expressed in Schwann cells and neurons during development, and its expression levels decline with age (Cosgaya et al., 2002). Both nerve crush injury and axotomy induce a strong upregulation of p75NTR in neurons and Schwann cells in distal nerve segments (Ernfors et al., 1989; Koliatsos et al., 1991; Saika et al., 1991; Syroid et al., 2000; Petratos et al., 2003; Figure 1). The role of p75NTR after injury is complex and partially contradictory (for a comprehensive review see Meeker and Williams, 2015), nonetheless, grafting of Schwann cells deficient for p75NTR impairs motor neuron survival after injury (Tomita et al., 2007). Moreover, remyelination is hampered in mice lacking p75NTR and nerve repair is delayed (Song et al., 2006; Chen et al., 2016). Thus, p75NTR likely has a pivotal role in supporting neuronal survival, nerve regeneration and remyelination after injury.

**Figure 2:**
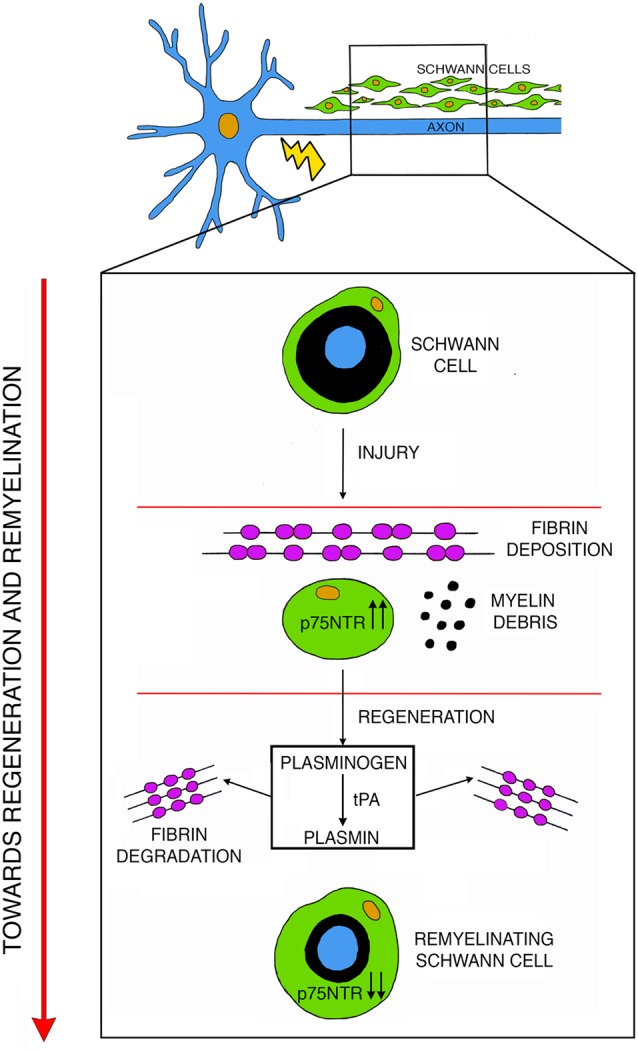
After injury, fibrin deposits inside the nerve while Schwann cells undergo de-differentiation and upregulate p75NTR. During active phases of regeneration p75NTR expression is diminished in remyelinating Schwann cells, and the induction of the plasminogen and of the tissue plasminogen activator (tPA)/plasmin cascade leads to fibrin degradation thus promoting remyelination. Modified with permission from Akassoglou et al. (2002).

In addition to the above-described roles, p75NTR also regulates fibrinolysis. Upon nerve injury, fibrin accumulates in the nerves to promote axon degeneration and Schwann cells dedifferentiation. However, to achieve efficient regeneration, fibrin has to be degraded in a process called fibrinolysis (Akassoglou et al., 2002). In injured nerves, accumulation of p75NTR in Schwann cells inhibits fibrinolysis thus maintaining Schwann cells in a un-differentiated state (Akassoglou et al., 2002; Sachs et al., 2007). During nerve regeneration, fibrin is degraded, p75NTR is downregulated and Schwann cells can re-differentiate to initiate remyelination (Akassoglou et al., 2000; Sachs et al., 2007; Figure 2). Thus, the biological role of p75NTR in axonal regeneration and remyelination is complex and probably depends on the relative contribution of its function as a regulator of cell death/survival and as an inhibitor of fibrin degradation.

Of note, the γ-secretase complex processes also axonal NRG1 type III (Bao et al., 2003, 2004). As already described, the extracellular processing of NRG1 type III prompts a forward signaling to initiate myelination (Hu et al., 2006; Willem et al., 2006; La Marca et al., 2011). The γ-secretase intracellular cleavage of axonal NRG1 type III, instead, generates a NRG1-ICD intracellular fragment, which activates the transcription of the lipocalin-type prostaglandin-D synthase (L-Pgds) gene (Trimarco et al., 2014). L-PGDS further induces a pro-myelinating pathway in Schwann cells controlling formation and maintenance of the myelin sheath (Trimarco et al., 2014). Given the importance of NRG1 in nerve regeneration, additional studies to clarify the contribution of NRG1-ICD and L-PGDS after injury could contribute to the understanding of the processes involved in nerve repair.

## Matrix Metalloproteinases

The matrix metalloproteinases (MMPs) are a family of zinc-dependent enzymes, which can degrade all components of the ECM (McCawley and Matrisian, 2001). At least 24 members, including collagenases, gelatinases, matrilysins, stromelysins and membrane-type MMPs belong to the MMPs family (Nagase et al., 2006). Proteolytic MMPs activity is necessary to regulate expression levels and function of ECM components and cell surface signaling receptors (Page-McCaw et al., 2007).

Among the various MMPs, MMP-2 and MMP-9 have been implicated in nerve development, and after injury (Platt et al., 2003). During development, MMP-2 and MMP-9 process dystroglycan to facilitate the formation of the dystroglycan receptor complex, a laminin receptor required for the correct formation of apposition and Cajal bands along myelinated fibers (Court et al., 2011).

Upon axotomy, MMP-9 mRNA levels are rapidly upregulated starting few hours after injury and peak at day 3 (La Fleur et al., 1996; Siebert et al., 2001; Hughes et al., 2002; Platt et al., 2003) while its activity increases already 12 h after axotomy, peaks around day 2 and returns to basal levels after 4 days (Siebert et al., 2001). In a model of crush nerve injury, MMP-9 localizes in myelinating Schwann cells, immune cells and endothelial cells (La Fleur et al., 1996; Shubayev and Myers, 2000; Hughes et al., 2002; Figure 1). During nerve regeneration, MMP-9 negatively controls Schwan cells proliferation (Chattopadhyay et al., 2007). Accordingly, nerves of mice lacking MMP-9 have increased numbers of post-mitotic Schwann cells, together with shorter internodes and nodal abnormalities (Kim et al., 2012). *In vivo* studies in mutants lacking MMP-9 indicate that MMP-9 activity during axonal degeneration is crucial to promote myelin sheath collapse and to prompt the blood-nerve-barrier breakdown allowing the influx of immune cells into the nerve (Shubayev and Myers, 2000; Chattopadhyay et al., 2007; Kobayashi et al., 2008). More recent studies have implicated also MMP-9, together with MMP-7, in regulating claudin proteins expression at the tight junction in nerves after crush injury, suggesting that high levels of MMPs might alter nerve physical barriers (Wang et al., 2018). Further, MMP-9 stimulates the rearrangement of the basal lamina (La Fleur et al., 1996; Hartung and Kieseier, 2000; Platt et al., 2003; Shubayev et al., 2006; Chattopadhyay et al., 2007; Kobayashi et al., 2008).

Interestingly, together with MMP-9, also its endogenous inhibitor TIMP-1 is upregulated in nerves early after injury (Kim et al., 2012); thus, it has been proposed that a fine balance between MMP-9 and TIMP-1 activity might regulate Schwann cells mitogenesis and maturation and control the molecular and structural assembly of myelin domains in remyelinated fibers to prompt a correct nerve repair (Kim et al., 2012). Thus, during early phases of axonal degeneration, a physiological inhibition of MMP-9 activity might be precisely coupled with its activation to coordinate an effective nerve repair.

## Plasminogen Activators and Related Proteases

The plasminogen activators (PAs), namely tissue PA (tPA) and urokinase PA (uPA), are key enzymes of the blood coagulation cascade implicated in several biological processes, including vascular and tissue remodeling, tumor development and progression (Mekkawy et al., 2014) and nervous system pathophysiology (Deryugina and Quigley, 2012; Thiebaut et al., 2018). These secreted proteases cleave the proenzyme plasminogen to turn it into its active form, plasmin (Collen, 1999; Rijken and Lijnen, 2009). Plasmin is a serine protease involved in processing a vast spectrum of molecules (Liotta et al., 1981), but its main substrate is fibrin (Bugge et al., 1996). Plasmin, in fact, degrades fibrin clots allowing scar resolution (Weisel and Litvinov, 2017). In addition, plasmin can activate other proteins, like MMPs (Keski-Oja et al., 1992; Murphy and Docherty, 1992; Zou et al., 2006) and growth factors (Saksela and Rifkin, 1990; Brauer and Yee, 1993; Munger et al., 1997). In the PNS, PAs activity is elevated during axonal outgrowth. PAs are secreted by primary neurons and Schwann cells in culture (Krystosek and Seeds, 1981, 1984) and their activity is localized in growth cones (Krystosek and Seeds, 1984). Moreover, the PA system is induced *in vivo* in embryonic DRG and motor neurons during axonal outgrowth (Seeds et al., 1992; Sumi et al., 1992). Interestingly, the PA system is potently induced also after peripheral nerve injury in mice (Siconolfi and Seeds, 2001a,b). Regeneration of peripheral nerves relies on the ability of axons to regrow in a very complex environment at the injury site, made of a structurally altered ECM, myelin and axon debris, surrounding tissues and infiltrating inflammatory cells. These adverse conditions represent an obstacle for axonal growth, thus regenerating neurites secrete PAs to dissolve cell-cell and cell-matrix adhesion. Accordingly, tPA and uPA mRNAs are promptly upregulated early after peripheral nerve injury in Schwann cells and neurons and their activity increases at the injury site up to 7 days post-crush (Akassoglou et al., 2000; Siconolfi and Seeds, 2001a; Figure 2). Moreover, recovery in knockout mice deficient for tPA, uPA and Plasminogen is significantly delayed after nerve injury, indicating that they are necessary for timely regeneration of peripheral nerves and the re-establishment of peripheral sensitivity (Siconolfi and Seeds, 2001b). Plasmin is therefore protective for nerve injury (Akassoglou et al., 2000) and this effect is linked to the proteolytic activity plasmin exerts on fibrin (Figure 2). Accordingly, the vast axonal damage observed in tPA and plasminogen knockout nerves correlates with excessive accumulation of fibrin. Interestingly, genetic and pharmacological depletion of fibrinogen, the fibrin molecular precursor, enhances regeneration in these mutants (Akassoglou et al., 2000). Thus, fibrin deposition exacerbates axonal injury and the physiological induction of the tPA/plasmin cascade after injury prompts fibrin removal and stimulates repair (Figure 2). Of note, the important role of this cascade has been described also in humans, as there is a direct correlation between the extent of fibrinolytic activity and the degree of regeneration in patients with peripheral neuropathies (Previtali et al., 2008; Rivellini et al., 2012).

In addition to the PA system, thrombin, another key enzyme of the blood coagulation system, and its inhibitor Nexin-1, are potently induced after sciatic nerve injury. In the blood coagulation cascade, thrombin converts fibrinogen into fibrin, its insoluble derivative (Weisel and Litvinov, 2017). Nexin-1, instead, a member of the large serpin superfamily, directly inhibits thrombin proteolytic activity (Cavanaugh et al., 1990; Gurwitz and Cunningham, 1990). In injured sciatic nerves, thrombin levels are elevated immediately after damage and reach their peak 1 day post-injury, declining after one week (Smirnova et al., 1996; Gera et al., 2016). On the contrary, upregulation of Nexin-1 in injured nerves occurs 6–9 days after thrombin induction (Meier et al., 1989; Smirnova et al., 1996) and is required to neutralize excessive thrombin proteolytic activity.

Collectively, these studies indicate that the proteases of the blood coagulation system and their regulators are essential to stimulate recovery in injured PNS and a precise balance between activation and inhibition of their proteolytic activity is required to correctly modulate nerve repair.

## Concluding Remarks

In the present review article, we presented a selected range of molecules and pathways involved in injured PNS. We specifically focused on extra cellular matrix and tissue modifiers and on growth factors’ processing. Several studies have demonstrated how crucial is the role of these enzymes in tissue remodeling in response to damage. Nonetheless, despite the extensive studies conducted thus far, additional work is required to better define both the cell autonomy and the time frame in which these molecules and pathways are mostly active. We thus think that clarifying these aspects could be beneficial to define new therapeutic strategies that, together with the development of conduit devices, could efficiently improve the regenerative process and prompt full functional recovery.

## Author Contributions

MP and CT searched PubMed for articles published up until August 2018. Both MP and CT discussed the topics, decided the outline of the review and wrote the manuscript.

## Conflict of Interest Statement

The authors declare that the research was conducted in the absence of any commercial or financial relationships that could be construed as a potential conflict of interest.
